# Ischemic stroke in anti-β2-glycoprotein I IgA-associated non-criteria antiphospholipid syndrome: a case report of arterial recanalization via antiplatelet therapy

**DOI:** 10.3389/fimmu.2025.1603526

**Published:** 2025-07-04

**Authors:** Jihong Liu, Yanru Chen

**Affiliations:** Neurology Department, The Second Affiliated Hospital of Chongqing Medical University, Chongqing, China

**Keywords:** antiphospholipid syndrome, seronegative antiphospholipid syndrome, anti-β2-glycoprotein I IgA, ischemic stroke, antiplatelet therapy, arterial recanalization, case report

## Abstract

**Background:**

The pathogenic potential of non-criteria antiphospholipid antibodies (aPLs), such as anti-β2-glycoprotein I (aβ2GPI) IgA, remains undefined. Additionally, the role of antiplatelet therapy in thrombotic antiphospholipid syndrome (APS) is controversial. Diagnosing APS is challenging when consensus aPLs are negative. Arterial recanalization via antiplatelet therapy in thrombotic APS has not been reported so far.

**Case presentation:**

A 65-year-old woman presented with acute basilar artery occlusion. Dual antiplatelet therapy (aspirin + clopidogrel) achieved complete arterial recanalization within 11 days. High-Resolution Magnetic Resonance Imaging excluded atherosclerosis, dissection, or arteritis. Cardiac evaluations ruled out cardioembolism. Laboratory tests revealed persistent isolated high-titer aβ2GPI IgA (>150 U/mL) with negative consensus aPLs. Thrombotic workup excluded hereditary or other secondary thrombophilia, supporting a diagnosis of non-criteria APS. Long-term aspirin monotherapy maintained vascular patency and prevented stroke recurrence over 5 years.

**Conclusion:**

This case highlights the thrombogenic role of aβ2GPI IgA in seronegative APS and demonstrates the potential efficacy of antiplatelet therapy, a strategy not prioritized in current APS guidelines favoring anticoagulation therapy, in reversing arterial occlusion and preventing recurrence in non-criteria APS. It underscores the need to re-evaluate diagnostic criteria and therapeutic strategy for seronegative APS with non-criteria aPLs.

## Introduction

Antiphospholipid syndrome (APS) is an autoimmune disorder characterized by thrombotic events or recurrent abortion. The diagnosis of APS relies on persistent moderate-to-high titers of “consensus” antiphospholipid antibodies (aPLs), including lupus anticoagulant (LA), anticardiolipin (aCL) IgG/IgM, and anti-β2-glycoprotein I (aβ2GPI) IgG/IgM (1). Although growing evidence suggests that non-criteria antibodies may independently contribute to thrombotic events, their diagnostic utility remains controversial, posing challenges for APS diagnosis when consensus aPLs are negative.

In patients with definite APS and first arterial thrombosis, treatment with vitamin K antagonists is recommended over treatment with low-dose aspirin only (2). This report describes a case of acute basilar artery occlusion in a patient with isolated high-titer aβ2GPI IgA and negative consensus aPLs. Strikingly, early dual antiplatelet therapy (DAPT) achieved complete arterial recanalization, and long-term aspirin monotherapy effectively prevented stroke recurrence over a 5-year follow-up. This case adds favorable evidence to the pathogenic role of aβ2GPI IgA in arterial thrombosis, highlights the potential efficacy of antiplatelet therapy in non-criteria aPLs-associated APS, challenges the conventional anticoagulation-centered paradigm, and provides novel insights into individualized management for this subgroup.

## Case presentation

A 65-year-old female was admitted to our hospital with a 2-day history of episodic dizziness, amaurosis, and loss of consciousness, accompanied by bilateral lower limbs weakness and drop attack, with spontaneous recovery within a few minutes. She experienced marked neurological deterioration upon admission, manifesting as vertigo, diplopia, dysarthria, and tetraparesis. The patient had a history of well-controlled hypertension and coronary heart disease. She denied diabetes mellitus, hyperlipidemia, atrial fibrillation, or smoking and alcohol use. Her BMI was 23.2kg/m^2^. Neurological examination revealed dysarthria, bilateral vertical nystagmus, right-sided one-and-a-half syndrome, left-sided central facial palsy, left-sided hemihypoesthesia, grade 4 muscle strength in bilateral upper limbs and grade 3 muscle strength in bilateral lower limbs, ataxia of both upper limbs, and a positive left-sided Babinski sign. The National Institutes of Health Stroke Scale (NIHSS) score was 12.

**Figure 1:**
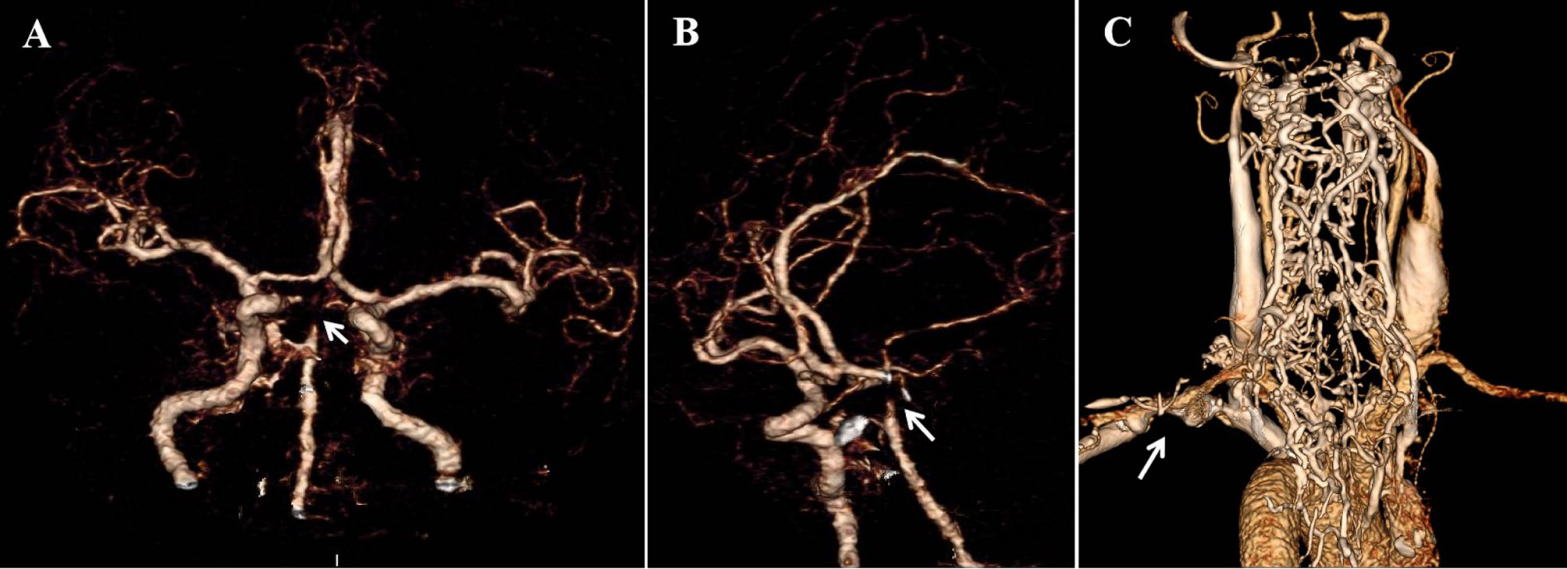
Emergency multimodal brain CT upon admission **(A, B)** CT angiography revealed severe stenosis and occlusion at the apex of BA and the P1 segment of bilateral PCA (white arrow), as shown in coronal **(A)** and oblique sagittal **(B)** views. **(C)** Occlusion of the left subclavian vein with retrograde flow in the jugular vein and perivertebral venous plexus.

Emergency multimodal brain CT imaging revealed no intracranial hemorrhage, but severe stenosis and occlusion at the apex of the basilar artery (BA) and the P1 segment of bilateral posterior cerebral arteries (PCA). Occlusion of the left subclavian vein was also observed, accompanied with retrograde flow in the jugular vein and perivertebral venous plexus (**Figure 1**). Emergency laboratory tests, including complete blood count, urinalysis, liver and renal function, electrolytes, coagulation profile, D-dimer, myocardial enzyme spectrum, and troponin, showed no notable abnormalities. Ischemic stroke was the initial diagnosis. The patient declined emergency endovascular intervention. DAPT with aspirin (100 mg/day) and clopidogrel (300mg loading dose followed by 75mg/day) was initiated upon admission, along with atorvastatin (40 mg/day), butylphthalide and edaravone. Blood pressure and blood glucose were closely monitored and controlled.

**Figure 2:**
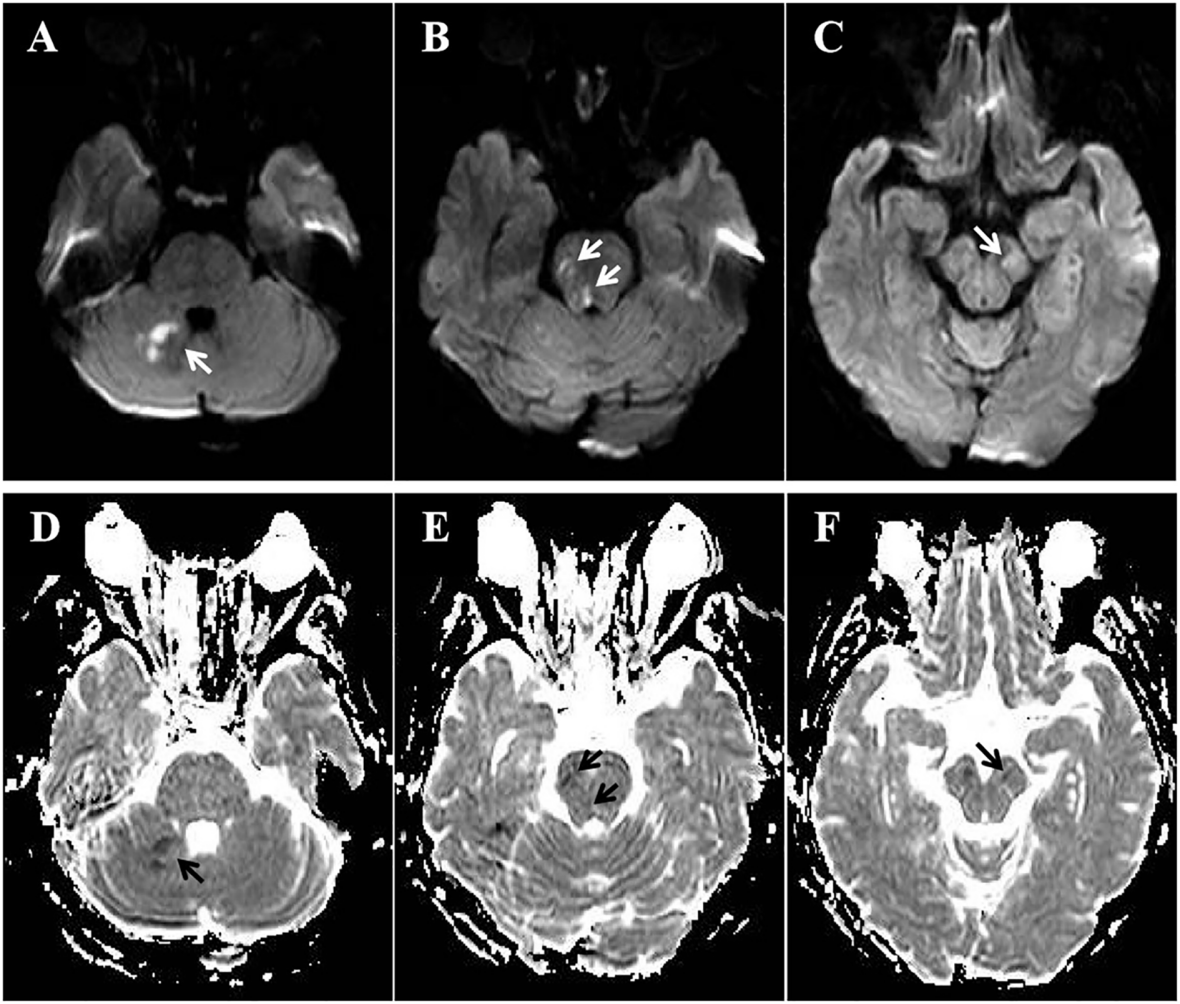
MRI on day 2. **(A-C)** Diffusion-weighted imaging showed acute infarction lesions in right cerebellum (**A**, white arrow), right pons (**B**, white arrow), and left midbrain (**C**, white arrow). **(D-F)** Apparent diffusion coefficient imaging revealed restricted diffusion in right cerebellum (**D**, black arrow), right pons (**E**, black arrows), and left midbrain (**F**, black arrow).

On day 2, the patient’s dizziness subsided, and the NIHSS score decreased to 10 as her bilateral lower limb muscle strength improved to grade IV. Magnetic resonance imaging demonstrated multiple infarct lesions in the brainstem and right cerebellum (**Figure 2**). Laboratory results revealed mild hyperlipidemia (TG 1.15mmol/L, TC 5.29mmol/L, LDLc 2.77mmol/L, HDL 1.34mmol/L), while thyroid function, glycated hemoglobin, homocysteine, and uric acid levels were normal. The ischemic stroke was classified as large artery atherosclerosis according to the TOAST classification. Other diagnoses included occlusion of the BA apex, stage 3 hypertension with extremely high cardiovascular risk, dyslipidemia, and occlusion of the left subclavian vein.

**Figure 3:**
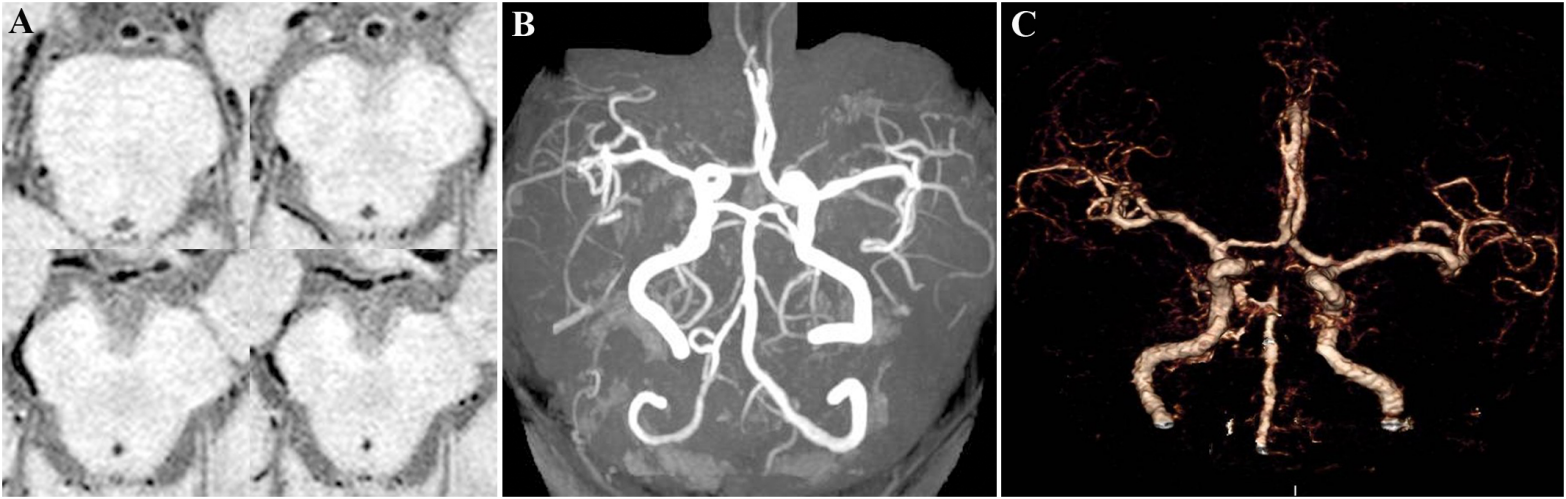
HRMRI of the cerebral vascular walls on day 11. **(A)** Axial vessel wall imaging revealed mild stenosis in BA and the P1 segments of bilateral PCA, with minor atherosclerotic plaques on the vessel walls. **(B)** Coronal 3D-TOF-MRA image showed complete recanalization of BA and the P1 segments of bilateral PCA compared with CT angiography upon admission **(C)**.

After treatment, the patient’s symptoms progressively improved, and the NIHSS score decreased to 6 on day 8. High-resolution magnetic resonance imaging (HRMRI) of the cerebral vascular walls on day 11 revealed mild stenosis in BA and the P1 segments of bilateral PCA, with minor atherosclerotic plaques on the vessel walls (**Figure 3**). Since the arteries had recanalized after short-term antiplatelet therapy and the atherosclerotic plaque burden was relatively low, it was assumed that the arteries occlusion was not caused by atherosclerosis. Further investigation of the patient’s medical history revealed no evidence of heart failure, tumors, arteriovenous thrombosis, connective tissue disorders, hematologic diseases, purpura, or any urine discoloration. She also denied recent infections, trauma, surgical procedures, or use of hemostatic agents, hematopoietic growth factors, glucocorticoids, oral contraceptives, or estrogen. There was no family history of thrombotic disorders or hereditary diseases. Cardiac evaluations, including echocardiography, 24-hour dynamic electrocardiogram, and contrast-enhanced transcranial Doppler, revealed no significant abnormalities. Tumor screening evaluations, including chest CT, abdominal ultrasound, gynecological and urinary tract ultrasounds, and tumor markers were unremarkable. Thromboelastography demonstrated a mildly elevated maximum amplitude (71.4 mm, normal range 50–70mm). Activities of protein C, protein S, antithrombin III, and factor VIII, along with erythrocyte sedimentation rate, immunoglobulin levels, antinuclear antibody profile, vasculitis antibodies, and anti-dsDNA antibodies, were all within normal reference ranges. LA (detected and evaluated according to the ISTH recommendations), serum aCL IgG/IgM/IgA, and aβ2GPI IgG/IgM were negative, while aβ2GPI IgA significantly increased (>150 U/ml, normal range 0–20 U/ml). IgG, IgM, IgA isotypes of aCL and aβ2GPI were analyzed with AESKULISA^®^ ELISA Test Kits provided by Aesku. Diagnostics GmbH & Co. KG (Wendelsheim, Germany). APS was highly suspected, thus aspirin and clopidogrel were discontinued, and low-molecular-weight heparin (4000 IU every 12 hours) was initiated and bridged to warfarin (2.5 mg/day), with a target international normalized ratio (INR) of 2-3.

**Figure 4:**
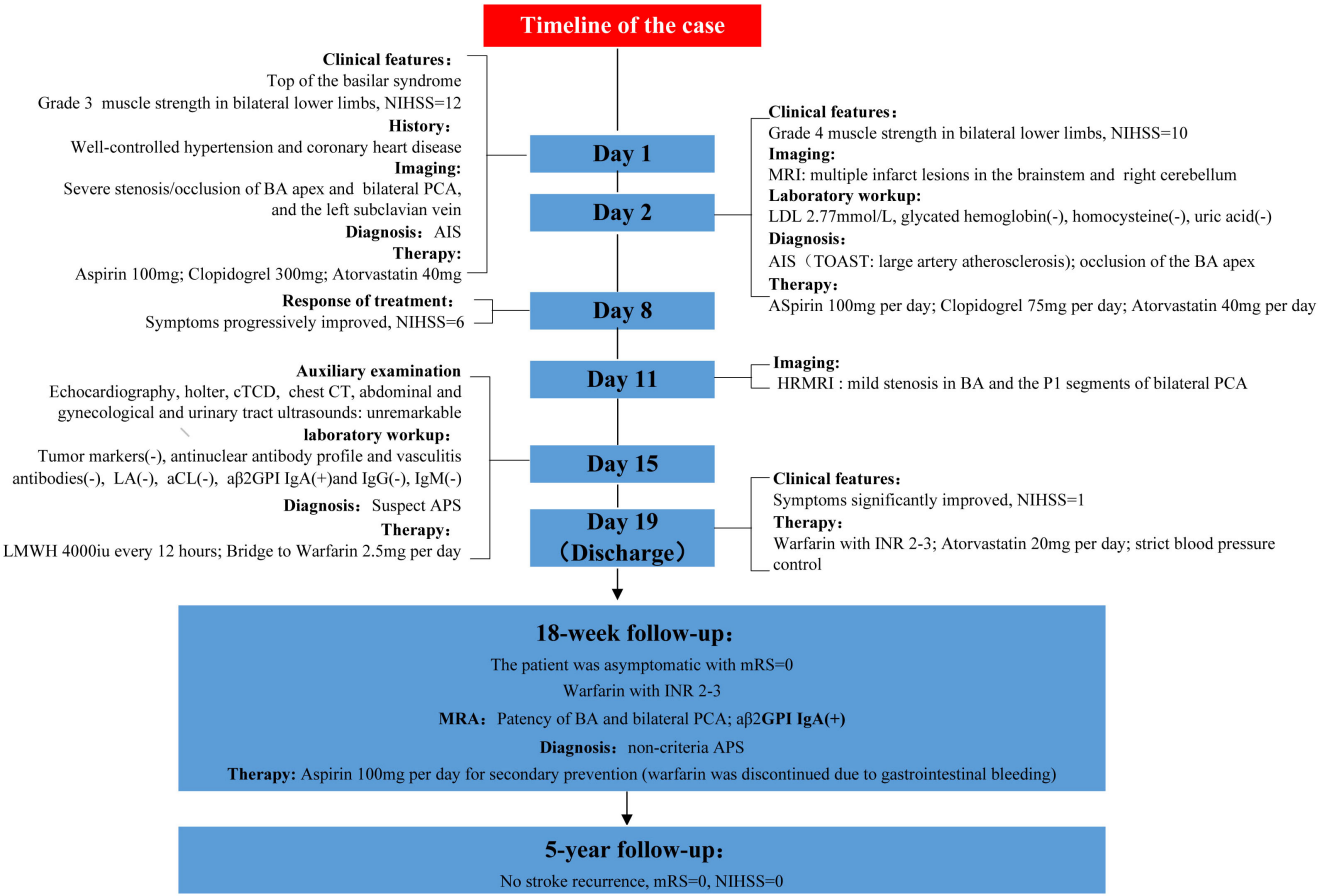
Timeline of the present case.

The patient’s NIHSS score was 1 upon discharge. Post-discharge treatment consisted of warfarin (INR 2–3), atorvastatin (20 mg/day), and strict blood pressure control (target <140/90 mmHg). At the 18-week follow-up, the patient was asymptomatic, with a modified Rankin score (mRS) of 0. Magnetic resonance angiography (MRA) demonstrated patency of BA, with mild stenosis of the P1 segment of bilateral PCA. Re-assessment of aPLs revealed a persistent high titer of aβ2GPI IgA (>400 U/ml, normal range 0–20 U/ml) with negative consensus aPLs, confirming the diagnosis of non-criteria APS and ischemic stroke secondary to the rare etiology. However, due to significant INR fluctuations (range 1.8-4.1) and non-severe gastrointestinal bleeding (confirmed by moderate anemia (hemoglobin 89 g/L, normal range 115–150 g/L) and a positive fecal occult blood test), warfarin was discontinued, and omeprazole along with rebamipide were initiated for gastroprotection. The patient’s CYP2C19 genotype indicated intermediate metabolism, suggesting clopidogrel resistance, thus aspirin (100 mg/day) was administered for secondary prevention under close clinical surveillance. The adjusted therapeutic regimen resulted in effective control of gastrointestinal bleeding and restoration of hemoglobin levels to 122 g/L Over 5 years of follow-up, no stroke recurrence or active gastrointestinal bleeding events occurred. The timeline of the case is shown in **Figure 4**.

## Discussion

Arterial recanalization after short-term antiplatelet therapy can be observed in various conditions, including vulnerable atherosclerotic plaques, arterial dissection, cardioembolism, and hypercoagulable state. In this patient, cerebral vessel wall HRMRI excluded vulnerable plaques, arterial dissection, and vasculitis, while cardiac evaluation ruled out cardioembolism. The concurrent BA thrombosis and subclavian vein occlusion strongly suggested hypercoagulability. Therefore, a comprehensive thrombophilia workup was performed, including both hereditary and acquired causes. To avoid missing seronegative APS (SN-APS), the aPLs profile was thoroughly evaluated, including non-criteria antibodies such as aβ2GPI IgA and aCL IgA. Laboratory testing revealed persistent high titer of aβ2GPI IgA despite negative LA, aCL antibodies, and aβ2GPI IgG/IgM. Although not meeting APS criteria, the comprehensive workup excluded hereditary thrombophilias and other acquired causes of hypercoagulability (medication-induced, malignancy, myeloproliferative disorders, hyperhomocysteinemia, paroxysmal nocturnal hemoglobinuria, thrombotic microangiopathy, macroglobulinemia, and vasculitis). These findings supported a diagnosis of non-criteria APS (3, 4).

The diagnostic and clinical significance of aβ2GPI IgA in APS remains controversial. Although aβ2GPI IgA is not currently included as diagnostic criteria for APS according to 2023 ACR/EULAR antiphospholipid syndrome classification criteria (1), emerging evidence indicates that aβ2GPI IgA can form high-affinity complexes with the β2GPI protein (5, 6). These aβ2GP1 IgA/β2GP1 immune complexes may deposit on vascular endothelium, inducing localized inflammation and upregulating platelet glycoprotein IIb/IIIa expression, thereby promoting platelet activation and thrombogenesis (7). Supporting this pathogenic mechanism, murine studies demonstrated that the injection of patient-derived aβ2GPI IgA induces femoral vein thrombosis *in vivo* (8). One of the biggest series of APS data from three different cohorts (Lupus in Minorities, LUMINA, the John Hopkins SLE cohort) showed that more than two-thirds of patients with isolated positive aβ2GPI IgA had APS clinical manifestations, and isolated positive aβ2GPI IgA was significantly associated with increased risk of arterial thrombosis and all thrombosis, even after adjusting for other risk factors of thrombosis (8, 9). Another seronegative APS (SN-APS) cohort also demonstrated the significant association between isolated positive aβ2GPI IgA and thrombotic events (6). Tortosa et al. reported that asymptomatic carriers of isolated positive aβ2GPI IgA (n=244) had significantly higher incidence of APS-events and arterial thrombosis than seronegative controls (n=221) in 5-year follow-up, with ischemic stroke emerging as the predominant arterial event in patients with aB2GP1 IgA (10). In a cohort of 245 consecutive ischemic stroke patients, aPLs were detected in 28% of cases, with aβ2GPI IgA being the most prevalent subtype (20%). Notably, aβ2GPI IgA contributed more significantly to stroke than other conventional risk factors such as hypertension or dyslipidemia (11). A population-based cohort study including 2427 participants found that the prevalence of any aPL at a single time point was 14.5%, and positive aCL IgA and aβ2GPI IgA were independently associated with future atherosclerotic cardiovascular disease (ASCVD) events (12). Accumulating evidence suggests that aβ2GPI IgA is an important independent risk factor for thrombosis, ischemic stroke, and atherosclerosis (5–14), especially among primary APS populations (3, 4, 7, 14). Although aβ2GPI IgA lacks sufficient diagnostic validation for inclusion in APS criteria, its clinical significance warrants consideration in specific scenarios. For patients exhibiting characteristic thrombotic events with negative consensus aPLs and exhaustive exclusion of alternative etiologies, persistent moderate-to-high titer of aβ2GPI IgA may justify a diagnosis of “non-criteria APS” or seronegative APS. In such cases, APS-targeted therapeutic management is clinically appropriate (3, 4, 7, 14–17).

The recommended management of APS-related thrombosis requires long-term anticoagulation therapy, typically with vitamin K antagonists (e.g., warfarin). However, the role of antiplatelet therapy in thrombotic APS remains controversial due to limited efficacy evidence (1, 2, 18). Retrospective studies suggest that antiplatelet therapy may be a viable alternative in patients who experience a single arterial event or anticoagulant intolerance. However, its preventive efficacy is significantly inferior to anticoagulation (19, 20). Notably, while low-molecular-weight heparin has demonstrated successful recanalization efficacy in APS patients with severe carotid artery stenosis/occlusion (21), no reported cases have shown arterial recanalization following antiplatelet therapy in APS-related thrombosis. In this case, complete recanalization of the occluded BA and bilateral PCA occurred following 11 days of DAPT, suggesting the potential efficacy of antiplatelet therapy in reversing non-criteria APS-associated arterial thrombosis. Warfarin, the guideline-recommended first-line therapy for secondary prevention, was discontinued due to significant INR variability and gastrointestinal bleeding. Although current guidelines do not recommend antiplatelet monotherapy for APS-associated arterial thrombosis, the observed vascular recanalization following DAPT suggested therapeutic efficacy of antiplatelet therapy in this patient. Given the presence of clopidogrel resistance, aspirin was presumed to be effective and empirically administered for secondary prevention under close clinical surveillance. Notably, the patient maintained stroke-free status for five years on aspirin monotherapy, highlighting the value of antiplatelet therapy in secondary prevention of arterial thrombosis caused by non-criteria antibodies (particularly aβ2GPI IgA) associated APS. The therapeutic response may reflect the distinct pathophysiology of aβ2GPI IgA-mediated thrombosis, characterized by platelet activation. Studies have shown that anti-β2GPI/β2GPI immune complexes bind to ApoER2 and glycoprotein Ibα, inducing platelet activation. Receptor cross-linking with anti-β2GPI further activates platelets, stimulating thromboxane A2 release. This process neutralizes the inhibitory effect of β2GPI on von Willebrand factor, thereby enhancing platelet adhesion and aggregation (7, 22). Platelet factor 4, a thrombogenic protein released by activated platelets, can form stable complexes with β2GPI in APS patients, and aβ2GPI antibodies binding to these complexes trigger p38 MAPK phosphorylation and subsequent thromboxane B2 production (23). Furthermore, platelet activation in APS was accompanied by upregulation of the mTORC2/Akt pathway and enhanced phosphorylation of SIN1 at threonine, and patient-derived aβ2GPI antibodies were shown to enhance platelet activation via this pathway (24). These findings underscore the critical role of platelets in the pathogenesis of aβ2GPI IgA-mediated thrombosis, which may exhibit different structural characteristics from that mediated by consensus aPLs, and potentially necessitates alternative treatment strategies. Nevertheless, generalization of these findings is limited by the inherent constraints of the single-case report, and larger prospective studies are warranted to validate the efficacy of antiplatelet therapy in non-criteria APS cohorts.

## Conclusion

This case provides compelling evidence for the thrombogenic potential of non-criteria antibodies, particularly aβ2GPI IgA. It is imperative to recognize and emphasize the potential role of these non-criteria antibodies in thrombosis pathogenesis. Notably, the observed response of aβ2GPI IgA-mediated arterial thrombosis to antiplatelet therapy without conventional anticoagulation in this case merits further evaluation in larger studies.

## Data Availability

The original contributions presented in the study are included in the article/supplementary material. Further inquiries can be directed to the corresponding author.

## References

[1] Barbhaiya M Zuily S Naden R Hendry A Manneville F Amigo MC . ACR/EULAR APS Classification Criteria Collaborators. 2023 ACR/EULAR antiphospholipid syndrome classification criteria. Ann Rheum Dis. (2023) 82:1258–70. doi: 10.1136/ard-2023-224609 37640450

[2] Tektonidou MG Andreoli L Limper M Amoura Z Cervera R Costedoat-Chalumeau N . EULAR recommendations for the management of antiphospholipid syndrome in adults. Ann Rheum Dis. (2019) 78:1296–304. doi: 10.1136/annrheumdis-2019-215213 PMC11034817 31092409

[3] Li S Bai Y Meng J Wang Q Tian X Li M . Prevalence and diagnostic value of non-criteria antiphospholipid antibodies for antiphospholipid syndrome in Chinese patients. Front Immunol. (2023) 14:1107510. doi: 10.3389/fimmu.2023.1107510 37122726 PMC10132625

[4] Celia AI Galli M Mancuso S Alessandri C Frati G Sciarretta S . Antiphospholipid syndrome: insights into molecular mechanisms and clinical manifestations. J Clin Med. (2024) 13:4191. doi: 10.3390/jcm13144191 39064231 PMC11277906

[5] Serrano M Morán L Martinez-Flores JA Mancebo E Pleguezuelo D Cabrera-Marante O . Immune complexes of beta-2-glycoprotein I and igA antiphospholipid antibodies identify patients with elevated risk of thrombosis and early mortality after heart transplantation. Front Immunol. (2019) 10:2891. doi: 10.3389/fimmu.2019.02891 31921152 PMC6935976

[6] Serrano M Martinez-Flores JA Norman GL Naranjo L Morales JM Serrano A . The igA isotype of anti-β2 glycoprotein I antibodies recognizes epitopes in domains 3, 4, and 5 that are located in a lateral zone of the molecule (L-shaped). Front Immunol. (2019) 10:1031. doi: 10.3389/fimmu.2019.01031 31134087 PMC6515947

[7] Cabrera-Marante O Rodríguez de Frías E Serrano M Lozano Morillo F Naranjo L Gil-Etayo FJ . The weight of igA anti-β2glycoprotein I in the antiphospholipid syndrome pathogenesis: closing the gap of seronegative antiphospholipid syndrome. Int J Mol Sci. (2020) 21:8972. doi: 10.3390/ijms21238972 33255963 PMC7730063

[8] Murthy V Willis R Romay-Penabad Z Ruiz-Limón P Martínez-Martínez LA Jatwani S . Value of isolated IgA anti-β2 -glycoprotein I positivity in the diagnosis of the antiphospholipid syndrome. Arthritis Rheumatol. (2013) 65:3186–93. doi: 10.1002/art.38131 PMC4048705 23983008

[9] Andreoli L Fredi M Nalli C Piantoni S Reggia R Dall’Ara F . Clinical significance of IgA anti-cardiolipin and IgA anti-β2glycoprotein I antibodies. Curr Rheum Rep. (2013) 15:343. doi: 10.1007/s11926-013-0343-1 23754504

[10] Tortosa C Cabrera-Marante O Serrano M Martínez-Flores JA Pérez D Lora D . Incidence of thromboembolic events in asymptomatic carriers of IgA anti ß2 glycoprotein-I antibodies. PloS One. (2017) 12:e0178889. doi: 10.1371/journal.pone.0178889 28727732 PMC5519006

[11] Naranjo L Ostos F Gil-Etayo FJ Hernández-Gallego J Cabrera-Marante Ó Pleguezuelo DE . Presence of extra-criteria antiphospholipid antibodies is an independent risk factor for ischemic stroke. Front Cardiovasc Med. (2021) 8:665741. doi: 10.3389/fcvm.2021.665741 34012984 PMC8126615

[12] Zuo Y Navaz S Liang W Li C Ayers CR Rysenga CE . Prevalence of antiphospholipid antibodies and association with incident cardiovascular events. JAMA Netw Open. (2023) 6:e236530. doi: 10.1001/jamanetworkopen.2023.6530 37014642 PMC10074226

[13] Selmi C De Santis M Battezzati PM Generali E Lari SA Ceribelli A . Anti-phospholipid antibody prevalence and association with subclinical atherosclerosis and atherothrombosis in the general population. Int J Cardiol. (2020) 300:209–13. doi: 10.1016/j.ijcard.2019.10.042 31757648

[14] El Hasbani G Uthman I . Lupus, antiphospholipid syndrome, and stroke: An attempt to crossmatch. Lupus. (2023) 32:593–602. doi: 10.1177/09612033231165151 36940089

[15] Pignatelli P Ettorre E Menichelli D Pani A Violi F Pastori D . Seronegative antiphospholipid syndrome: refining the value of “non-criteria” antibodies for diagnosis and clinical management. Haematologica. (2020) 105:562–72. doi: 10.3324/haematol.2019.221945 PMC7049333 32001534

[16] Pérez D Tincani A Serrano M Shoenfeld Y Serrano A . Antiphospholipid syndrome and IgA anti-beta2-glycoprotein I antibodies: when Cinderella becomes a princess. Lupus. (2018) 27:177–8. doi: 10.1177/0961203317738227 29067871

[17] Zhao JL Shen HL Chai KX Yang CD Zhao Y . Recommendations for management of antiphospholipid syndrome in China. Zhonghua Nei Ke Za Zhi. (2022) 61:1000–7. doi: 10.3760/cma.j.cn112138-20211222-00907 36008292

[18] Knight JS Branch DW Ortel TL . Antiphospholipid syndrome: advances in diagnosis, pathogenesis, and management. BMJ. (2023) 380:e069717. doi: 10.1136/bmj-2021-069717 36849186

[19] Jackson WG Oromendia C Unlu O Erkan D DeSancho MT . Antiphospholipid Syndrome Alliance for Clinical Trials and International Networking. Recurrent thrombosis in patients with antiphospholipid antibodies and arterial thrombosis on antithrombotic therapy. Blood Adv. (2017) 1:2320–4. doi: 10.1182/bloodadvances.2017008185 PMC5729618 29296881

[20] Manning JE Arachchillage DJ . Dilemmas in the diagnosis and management of antiphospholipid syndrome. J Thromb Haemost. (2024) 22:2156–70. doi: 10.1016/j.jtha.2024.03.027 38705387

[21] Xiao F Tian X Wang XF . Antiphospholipid syndrome causing reversible internal carotid artery thrombosis. Lancet. (2018) 391:2641. doi: 10.1016/S0140-6736(18)31327-8 30070223

[22] Yang L Guo R Liu H Chen B Li C Liu R . Mechanism of antiphospholipid antibody-mediated thrombosis in antiphospholipid syndrome. Front Immunol. (2025) 16:1527554. doi: 10.3389/fimmu.2025.1527554 40181965 PMC11966034

[23] Sikara MP Routsias JG Samiotaki M Panayotou G Moutsopoulos HM Vlachoyiannopoulos PG . {beta}2 Glycoprotein I ({beta}2GPI) binds platelet factor 4 (PF4): implications for the pathogenesis of antiphospholipid syndrome. Blood. (2010) 115:713–23. doi: 10.1182/blood-2009-03-206367 19805618

[24] Tang Z Shi H Chen C Teng J Dai J Ouyang X . Activation of platelet mTORC2/akt pathway by anti-β2GP1 antibody promotes thrombosis in antiphospholipid syndrome. Arterioscler Thromb Vasc Biol. (2023) 43:1818–32. doi: 10.1161/ATVBAHA.123.318978 37381985

